# Curriculum Innovation: Neuro Day: An Innovative Educational Intervention to Enhance Interest in Neurology for Medical Trainees

**DOI:** 10.1212/NE9.0000000000200207

**Published:** 2025-04-11

**Authors:** Jessica Frey, Brandon Chase Neeley, Gabriella Casinelli, Benjamin Paserba, Delanie Talkington, Patrick Sheehan, James W. Lewis, Bruce Palmer, Anna Lama, Amelia Adcock, Eric Seachrist, Gauri V. Pawar, Ann Marie Murray

**Affiliations:** 1 Department of Neurology, Rockefeller Neuroscience Institute, West Virginia University, Morgantown; and; 2 Department of Medical Education, West Virginia University, Morgantown.

## Abstract

**Introduction and Problem Statement:**

Limited or delayed exposure to neurology decreases interest and reduces the likelihood of pursuing neurology as a career for medical trainees. Educational events that strengthen student-to-patient interactions may help dispel misinformation about neurologic treatments and outcomes. Innovative educational strategies such as the use of multimedia to teach the neurologic examination, incorporation of real patients, and integration of clinical neurology mentors were combined to create Neuro Day, an educational event designed to pique neurology interest for first-year medical students.

**Objectives:**

The aim of this intervention was to provide early neurology exposure to medical students to increase interest in neurology and interest in pursuing neurology for residency, improve perceptions of neurologic disease and treatment, and enhance neuroscience knowledge, as well as to improve physician-educator enjoyment of teaching clinical neurology.

**Methods and Curriculum:**

In 2023 and 2024, first-year medical students participated in Neuro Day. In part 1, students used an interactive video module to review neurology examination and pathophysiology. In part 2, student groups rotated to stations in a mock patient rounds format. Stations included patient-educators with real neurologic diseases paired with a physician-educator who moderated the discussion of the patient experience, pathophysiology, and examination findings. Students, patient-educators, and physician-educators were surveyed before and after Neuro Day. Statistical analysis included the paired *t* test with Cohen *d*, binomial test for difference in proportions, and thematic analysis.

**Results and Assessment Data:**

After Neuro Day, 108 of 115 (94%) and 98 of 103 (95%) first-year medical students and 10 of 13 physician-educators (77%) completed both presurvey and postsurvey, revealing the following: (1) increased interest in neurology (*d* = 0.27–0.68), (2) increased interest in pursuing neurology for residency (*d* = 0.26–0.38), (3) improved perception of patient outcomes (*d* = 0.45–0.67) and treatment options (*d* = 0.87–1.09), (4) 35.5% improvement in knowledge quiz scores (95% CI 0.218–0.492, *p* < 0.001), and (5) improved professional fulfillment in physician-educators (*d* = 1.20). Thematic analysis revealed that Neuro Day helped to humanize medicine, reinforce course content, and positively change perceptions.

**Discussion and Lessons Learned:**

Neuro Day is a feasible teaching paradigm to increase interest in neurology, reshape perceptions about neurology, improve confidence in performing a neurologic examination, reinforce neuroscience lecture content, encourage more students to pursue neurology residency, and improve physician-educator professional fulfillment.

## Introduction and Problem Statement

Developing innovative strategies to engage medical students early in their education is an important way to instill excitement and encourage interest in the field of neurology. Within the past 30 years, morbidity attributed to a neurologic cause has increased by 15% and mortality secondary to a neurologic illness has increased by 39%.^1^ Despite this, there are lower rates of students choosing to specialize in neurology, with 9% neurology residency spots unfilled in 2020 and an estimated 19% shortage in neurologists predicted to occur by 2025.^2^ There are many contributing factors to the lack of interest in neurology, including how neuroscience courses are taught, the outward perception of neurologists, stigma associated with conditions that may be challenging to treat, the complexity of neuroanatomy, lack of exposure to clinical neurology, and a fear of studying neurology termed “neurophobia.”^3^ In addition, neurophobia levels often remain stable throughout medical school, suggesting that early preconceptions have a large impact on trainees' beliefs about the specialty.^4,5^ Despite this, neurology is not a required core clerkship at more than 50% of medical schools, leading to limited exposure and diminished interest in the field.^6^ Studies have consistently demonstrated that most of the medical students who choose to pursue neurologic-related residency programs attended a school with a mandatory neurology clerkship.^6^ Other factors that lead trainees to select a neurologic-related residency training program include a required neurology clerkship in the third year as opposed to the fourth year, a 4-week or longer neurology clerkship, and availability of neurology-related electives such as pediatric neurology and neurosurgery.^7^

At our institution, the neurology clerkship is only 2 weeks long, truncating the amount of neurology exposure. In addition, there has been variability over the years as to whether the neurology clerkship is mandatory in the third year, with many medical students waiting until their fourth year to take the neurology clerkship.

Several strategies have been proposed in the literature to ensure adequate exposure, including early exposure to neuroscience (ideally in the first year of medical school), accessibility of neurology mentors, integration of clinical neurologists to teach the neuroscience curriculum, demonstration of neurology-related procedures, and inclusion of medical students in neurology research projects.^4,6,8^ At our institution, Neuro Day was designed as an early clinical exposure event for first-year medical students to increase interest in neurology in the short term and increase pursuance of a neurology career in the long term.

## Objectives

In 2019, our group piloted a “Neuro Day” experience for first-year medical students. The results of this pilot intervention demonstrated a significant increase in neurology interest, increase in perceived neurology knowledge, and reduction in fear of studying neurology.^9,10^ Based on the feedback from that initial pilot study, Neuro Day was implemented for first-year medical students on an annual basis. For students, the objectives are to (1) increase interest in neurology, (2) improve perceptions about neurology, (3) improve exposure to patients and access to neurology mentors early in medical training, (4) enhance knowledge taught in the neuroscience course, and (5) increase pursuance of neurology as a future career. For patient-educators, the objectives are to (1) improve understanding of their neurologic illness and (2) reframe their neurologic illness in a positive manner. For physician-educators, the objectives are to (1) improve comfort and enjoyment of teaching clinical neurology and (2) demonstrate the impact of patients as teachers.

## Methods and Curriculum

Neuro Day is a mandatory 4-hour educational intervention for first-year medical students midway through the core neuroscience block that has taken place annually since 2019, with social distancing and virtual modifications during academic years affected by coronavirus disease 2019 (COVID-19). In 2023 and 2024, Neuro Day was reintroduced using the in-person format.

### Standard Protocol Approvals, Registrations, and Participant Consents

West Virginia University Institutional Review Board approval (protocol 2302732165) was obtained for the distribution of surveys and implementation of semistructured interviews for the students, patient-educators, and physician-educators. Written informed consent was waived for all 3 participating groups. Although the educational event was mandatory for medical students, the surveys and semistructured interviews were optional. Patient-educators and physician-educators participated on a volunteer basis, and surveys were optional.

### Pre–Neuro Day

Before the Neuro Day event, medical students were asked to watch an interactive video demonstrating proper neurologic examination techniques, which included clickable hyperlinks explaining the underlying physiologic reasons for each part of the examination. For example, in the cranial nerves section, students could watch how to check for the corneal reflex and click a link to explore the physiologic reasons for the efferent and afferent pathways involved. The interactive aspects of the video allowed students to further explore sections they found challenging or particularly interesting. Before Neuro Day, students filled out a brief worksheet based on their interaction with the video tutorial for class credit, covering the most common and clinically relevant neurologic examination techniques.

The day before the event, students also completed an 8-question multiple-choice pre-event (and later postevent) knowledge quiz to assess their objective neurology knowledge, during regularly scheduled class time. The knowledge quiz was developed in collaboration with the neuroscience core curriculum faculty and clinical neurology faculty and residents, and questions covered neuroanatomy with clinical correlation. Face validity was established by having the neuroscience faculty and clinical neurology faculty and residents fill out the quiz and provide feedback about question stems and answer choices. Revisions were made until the questions were an accurate reflection of the neuroscience course material.

### Neuro Day

Using a mock patient rounds format, small groups of medical students (n = 10–12) rotated between rooms to visit 10 different stations, spending approximately 18 minutes per room with 2 minutes for rotation (eFigure 1). Roughly 8–9 stations consisted of patient volunteers with real neurologic diseases (Table 1), in which the patients shared their personal experience with their disease followed by a neurology physician (“physician-educator”) discussing the pathophysiology and highlighting clinically relevant examination findings. The students already had exposure to most of these examination techniques in the pre–Neuro Day interactive video. During the Neuro Day event, these examination techniques and their clinical application were reinforced. At the end of each session, students were allowed to interact with the patient, including asking them questions and observing and/or practicing disease-specific clinical examination techniques. Techniques included standard cranial nerve tests; reflexes and strength testing; and special techniques such as eliciting bradykinesia, cogwheel rigidity, fatigable weakness, myotonia, and pathologic reflexes including Hoffman sign and Babinski reflex. At the remaining 1–2 stations, a clinical neurologist supervised practicing neurologic examination techniques on each other.

**Table 1:** List of Patient-Educator Diagnoses by Year

Station number	2023	2024
1	Parkinson disease	Parkinson disease status/post deep brain stimulation
2	Stroke status/post hemicraniectomy	Stiff person syndrome
3	Cerebellar ataxia	Charcot-Marie-Tooth syndrome
4	Essential tremor status/post deep brain stimulation	Essential tremor status after deep brain stimulation
5	Epilepsy status/post responsive neurostimulation	Epilepsy status/post interstitial thermal therapy
6	Multiple sclerosis	Multiple sclerosis
7	Strokes and seizures secondary to marantic endocarditis	Huntington disease
8	Transverse myelitis	Transverse myelitis
9	Central nervous system–focused examination	Chronic inflammatory demyelinating polyneuropathy
10	Peripheral nerve–focused examination	Peripheral nerve–focused examination

There were several key differences between the 2019 pilot intervention and the current Neuro Day model, based on student and patient-educator feedback. The current model replaced the 1-hour pre-encounter lecture with extended small group discussions during the 4-hour rotation schedule. In addition, patient station time increased from 10 minutes per room in 2019 to 18 minutes per room in 2023 and 2024, allowing for more patient-student interaction. Furthermore, patient-educators and physician-educators were surveyed about their experiences participating in the Neuro Day event.

### Outcome Measures

Student surveys included questions graded on a Likert scale regarding interest in neurology and overall experience of Neuro Day (1 = strongly disagree and 5 = strongly agree), perceptions of neurology (1 = strongly disagree and 4 = strongly agree), experiences related to neurology exposure (1 = extremely low and 5 = extremely high), teaching strategies for neurology (1 = not helpful and 4 = extremely helpful), and interest pursuing particular residency specialties (1 = no interest and 5 = high interest). Patient-educators were surveyed on a Likert scale regarding their comfort level with teaching others about their diagnosis (1 = strongly disagree; and 4 = strongly agree). Physician-educators were surveyed using a Likert scale regarding their own medical knowledge, comfort level teaching neurology, professional fulfillment, and perception of patients with neurologic disease (1 = strongly disagree and 5 = strongly agree). Free-response sections were included in all surveys. All surveys are available in eAppendix 1.

During the post–Neuro Day luncheon with patient-educators and students, an announcement was made that semistructured interviews were occurring as an option to provide feedback on the Neuro Day event. The semistructured interviews were conducted by one of the physician-educators with open-ended questions such as “What did you like most/least about Neuro Day” and “How did the Neuro Day experience change your perspective on different neurologic conditions?” Interviews were audio-recorded and transcribed.

### Post–Neuro Day

Two days after the event during regularly scheduled class time, students completed a postevent objective knowledge quiz, with the same 8 questions as the pre-event quiz. They received credit for completion and an extra credit point if their score stayed the same or improved from the pre-event quiz.

### Statistical Analysis

Paired *t* tests were used for statistical analysis of surveys and objective quiz scores, and Cohen *d* was used to determine the effect size. In addition, responses were separated into binary categories with “agree” or “strongly agree” representing high interest/agreement and other answer choices representing low interest/agreement. A binomial test was used to calculate the difference in proportions of interest/agreement in each category before and after Neuro Day. Using data from the National Resident Matching Program (NRMP), the number of students matching into a neurologic-related residency program was also explored.

Thematic analysis was used to analyze the qualitative data collected. Semistructured interviews, of which the questions were developed before collection of the quantitative results, were audio-recorded and transcribed in full. Answers to free-response portions of the surveys were transcribed. Using an inductive approach, the authors (J.F., P.S., and D.T.) highlighted and selected a variety of discrete, embedded, and longer quotations from these transcriptions. Keywords were selected from these quotations to develop codes and eventually themes. Transcriptions were reviewed multiple times until all authors agreed that no further themes emerged. To reduce subjectivity, quotations that diverged from the developing themes were actively sought out. Themes were revised and finalized.

### Data Availability

Deidentified data and study protocol are available on request.

## Results and Assessment Data

In 2023 and 2024, there were 108 of 115 (94%) and 98 of 103 (95%) medical students who completed both a presurvey and postsurvey, respectively. The impact of Neuro Day on medical students' reaction, knowledge, behavior, and downstream results is summarized further. Complete data analysis can be accessed in the supplemental files in eAppendix 2 (eTables 1–6).

### Medical Students—Reaction: Overall Neuro Day Experience

**Figure 1:**
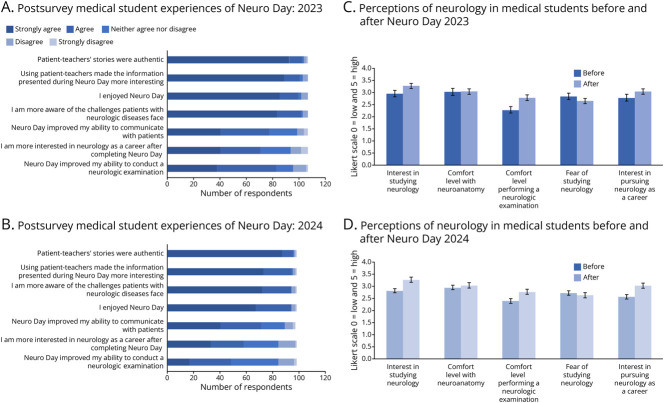
Medical Student Reaction to Neuro Day Overall medical student experience of Neuro Day in 2023 (A) and 2024 (B). Medical student perception of neurology before and after Neuro Day in 2023 (C) and 2024 (D).

Most of the respondents (>90%) agreed or strongly agreed that they enjoyed Neuro Day, the patient-educators’ stories were interesting, and they became more aware of the challenges faced by patients with neurologic diseases and felt that the patient-educators were authentic (Figure 1, A and B).

### Medical Students—Reaction: Perception of Neurology

Analysis with the *t* test and corresponding Cohen *d* revealed a statistically significant increased interest in neurology (2023: *p* = 0.003, *d* = 0.27, small effect; 2024: *p* < 0.001, *d* = 0.68, medium effect), improvement in comfort level performing a neurologic examination (2023: *p* < 0.001, *d* = 0.59, medium effect; 2024: *p* < 0.001, *d* = 0.44, medium effect), and interest in pursuing neurology as a future career (2023: *p* = 0.023, *d* = 0.26, small effect; 2024: *p* < 0.001, *d* = 0.38, small effect). Neuro Day had no impact on comfort level with neuroanatomy (Figure 1, C and D). Binomial testing indicated that the proportion of medical students interested in studying neurology significantly increased from 21.1% to 38.9% in 2024 (95% CI 0.049–0.309, *p* = 0.004) and the proportion of students interested in pursuing a career in neurology statistically increased from 19% to 30.1% and 13.7% to 25.3% in each year, respectively (2023: 95% CI 0.006–0.227, *p* = 0.032; 2024: 95% CI 0.003–0.228, *p* = 0.022).

There were several statistically significant changes in how students perceived neurology after Neuro Day in both 2023 and 2024. Scores related to limited exposure to neurology (2023: *p* < 0.001, *d* = 0.54, medium effect; 2024: *p* < 0.001, *d* = 0.53, medium effect), limited treatment options for patients with neurologic disease (2023: *p* < 0.001, *d* = 0.87, large effect; 2024: *p* < 0.001, *d* = 1.09, large effect), and patients with neurologic disease having poor outcomes (2023: *p* < 0.001, *d* = 0.45, medium effect; 2024: *p* < 0.001, *d* = 0.67, medium effect) all significantly decreased after Neuro Day across both years. In 2024, there was also a statistically significant decrease in medical students not needing to know neurology in the future (*p* = 0.001, *d* = 0.40, small effect). Similarly, binomial testing indicated that the proportion of medical students believing that they had limited exposure to neurology decreased from 55.6% to 34.3% and 51.0% to 32.7% in 2023 and 2024, respectively (2023: 95% 0.080–0.344, *p* < 0.001; 2024: 95% CI 0.046–0.322, *p* = 0.005); the proportion of students believing that there are limited treatment options significantly decreased from 57.4% to 21.3% and 55.1% to 14.3% in 2023 and 2024, respectively (2023: 95% CI 0.221–0.480, *p* < 0.001; 2024: 95% CI 0.275–0.541, *p* < 0.001); and the proportion of medical students believing that neurology patients have poor outcomes significantly decreased from 47.2% to 25.0% and 44.9% to 20.4% in 2023 and 2024, respectively (2023: 95% CI 0.086–0.341, *p* < 0.001; 2024: 95% CI 0.114–0.376, *p* < 0.001). There were no statistically significant differences in beliefs regarding neurology having a difficult reputation or in access to neurology mentors.

### Medical Students—Reaction: Teaching Strategies

In both 2023 and 2024, the most highly rated teaching method as a helpful strategy was through patient encounters. After Neuro Day, case discussions (2023: *p* = 0.027, *d* = 0.33, small effect; 2024: *p* < 0.001, *d* = 0.46, medium effect), clinical skill demonstrations (2023: *p* = 0.027, *d* = 0.34, small effect; 2024: *p* = 0.047, *d* = 0.23, small effect), and patient encounters (2023: *p* < 0.001, *d* = 0.64, medium effect; 2024: *p* < 0.001, *d* = 0.42, small effect) were rated as being statistically significantly more helpful. In 2024, access to neurology mentors was also rated as being statistically significantly more helpful (*p* < 0.001, *d* = 0.40, small effect) and the proportion of students identifying mentorship as an important teaching method significantly increased from 70.4% to 87.8% (95% CI 0.060–0.287, *p* = 0.001).

### Medical Students—Reaction: Interest in Neurology as a Career

**Figure 2:**
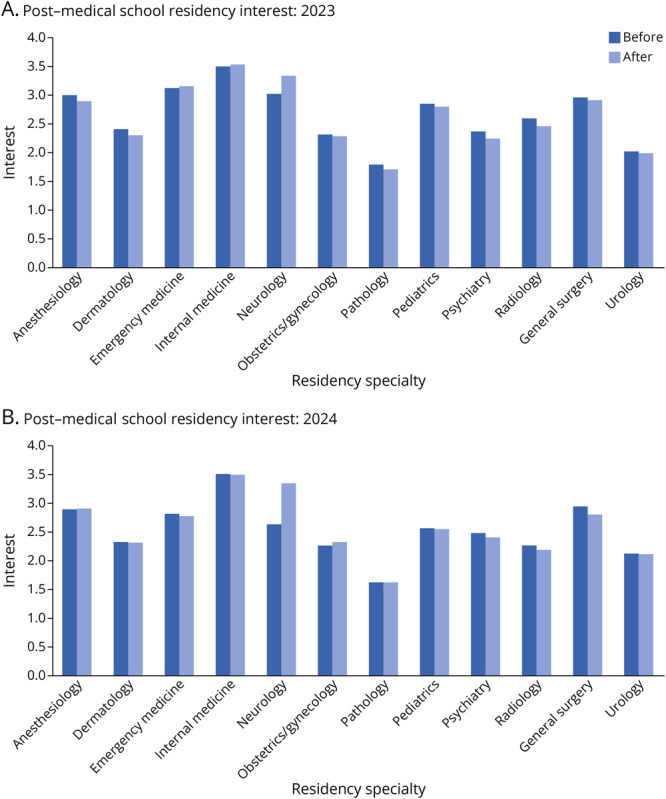
Residency Interest Medical student interest in pursuing various residency specialties before and after participation in Neuro Day in 2023 (A) and 2024 (B).

Internal medicine was ranked as the specialty that medical students were most interested in pursuing for residency before their participation in Neuro Day. After Neuro Day, there was a statistically significant increase in medical student interest in pursuing neurology for residency (2023: *p* = 0.034, *d* = 0.27, small effect; 2024: *p* < 0.001, *d* = 0.68, medium effect), whereas there were no statistically significant differences for any other residency specialty. The proportion of medical students interested in pursuing neurology residency after Neuro Day also significantly increased from 36.2% to 51.4% and 21.0% to 51.0% in 2023 and 2024, respectively (2023: 95% CI 0.018–0.287, *p* = 0.013; 2024: 95% CI 0.171–0.441, *p* < 0.001). After Neuro Day, neurology became the second most popular field to pursue, second only to internal medicine (Figure 2, A and B).

### Medical Students—Knowledge

Students who submitted the 8-question knowledge-based quiz both before and after Neuro Day (n = 87 of 103) were included in the analysis. There was a significant improvement in average quiz scores from 51.6% to 87.1% correct after Neuro Day (95% CI 0.218–0.492, *p* < 0.001).

### Medical Students—Behavior

**Figure 3:**
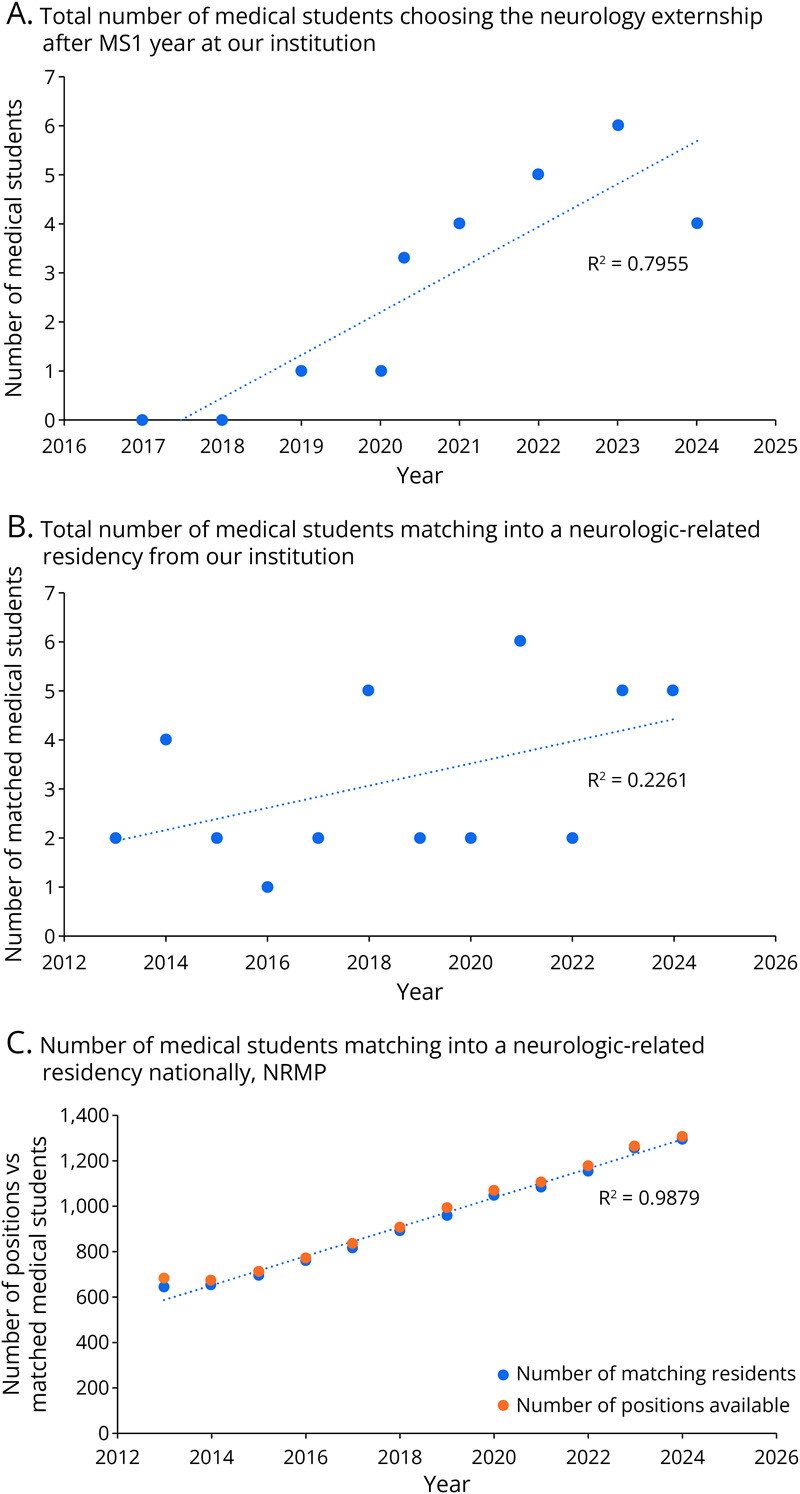
Neurologic-Related Fields as a Future Career (A) Number of medical students per year at our institution selecting an optional, volunteer-based neurology externship in the summer between the first and second year of medical school. (B) Number of medical students per year at our institution choosing to match into a neurologic-related residency (child neurology, neurology, neurosurgery, neurodevelopmental disabilities) on graduation. (C) Students per year matching into a neurologic-related residency nationally and the corresponding number of positions available per year, according to National Residency Matching Program data.

Between the first and second year of medical school at our institution, medical students have the opportunity to take elective “externships” that are approximately 4 weeks long, in a chosen specialty of interest. Apart from years that in-person learning was affected by the COVID-19 pandemic, more students chose to take the neurology externship in the years after Neuro Day was introduced to the first-year medical student curriculum (*R*^2^ = 0.80) (Figure 3A). Note that in 2019, the summer immediately after the first Neuro Day, the neurology externs had been selected before being exposed to Neuro Day.

### Medical Students—Downstream Results

The first Neuro Day event at our institution was held in 2019, which means that the first graduating medical student class that was exposed to the Neuro Day event would have graduated in 2022. Per year, there have been gradually more total number of graduating medical students (*R*^2^ = 0.24) matching into a neurologic-related residency (including neurology, neurosurgery, pediatric neurology, or neurodevelopmental disabilities) after Neuro Day was implemented at our institution (Figure 3B). From 2022 to 2024, there was an average of 4 medical students per year matching into a neurologic-related residency, whereas before that, the average was 2.89 students per year. NRMP data demonstrate that there is a corresponding increase in the number of neurologic-related residency positions each year (*R*^2^ = 0.99) and, nationally, these spots are being filled 94%–99% (Figure 3C).

Since 2021, our institution has offered a fast-track curriculum in which a student can apply to complete an abridged 3-year medical school program followed by entering residency in their specialty of choice. This fast-track curriculum has been highly competitive, given the expedited nature and overall cost savings, and students apply in the beginning of their second year. The residency programs that offer the fast-track option at our institution include anesthesiology, emergency medicine, family medicine, internal medicine, neurology, obstetrics/gynecology, pathology, pediatrics, and psychiatry. Of these, neurology is equally competitive at recruiting students. In fact, there has been such a draw for students to pursue the neurology fast-track option that we have actually had to turn away several applicants each year because there are only 2 spots allocated.

### Patient-Educators

In 2023, 6 of 8 patient-educators (75%) and in 2024, 6 of 9 patient-educators (67%) completed surveys. There were no statistically significant differences in patient-educator perceptions of their neurologic diagnosis. Across both years, all patient-educators rated highly their enjoyment of teaching others (100% agreed or strongly agreed) and gave low ratings for any negative feelings about sharing their neurologic condition. In the free-response section, gratitude was a common theme, with comments such as “This experience has forced growth in areas of my life that I am grateful for” and “Wonderful experience sharing my story.”

### Physician-Educators

**Figure 4:**
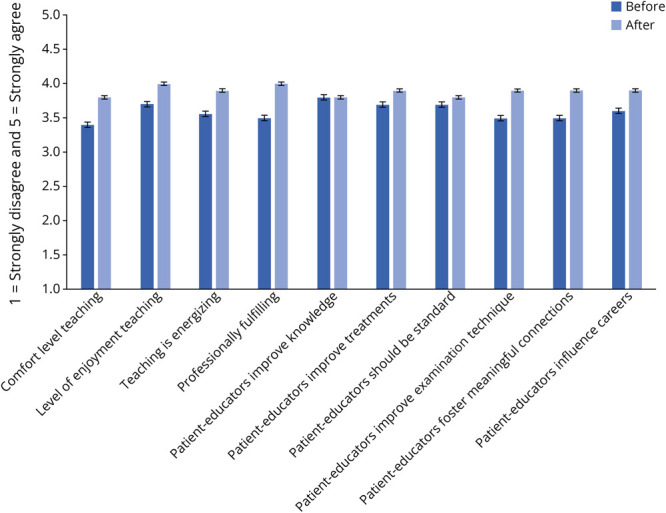
Physician-Educator Perceptions Impact of Neuro Day on physician-educator clinical teaching experience before and after Neuro Day in 2024.

In 2024, the physician-educators were also surveyed before and after Neuro Day about their experiences. Four faculty members (average postgraduate training years = 9.75 years) and 9 residents (postgraduate year [PGY] 2: 1; PGY3: 3; PGY4: 5) participated. Ten of 13 (77%) completed both presurvey and postsurvey. Results showed a statistically significant increase in professional fulfillment (*p* = 0.015, *d* = 1.20, large effect). There were no statistically significant differences in a variety of other domains, including enjoyment of teaching and the value of patient-educators for teaching neurology concepts to trainees (Figure 4). In the free-response section, the value of Neuro Day to the physician-educator was acknowledged with statements such as “[Neuro Day] is a lot of fun and helps me to learn as well” and “Wonderful experience as an educator to hear a patients experience through diagnosis and treatment, providing me a new perspective.”

### Thematic Analysis

A total of 21 students, in small groups of approximately 4–5, participated in the semistructured interviews. Their interviews were transcribed along with the responses to the free-response section in the surveys. Thematic analysis of these transcriptions identified multiple keywords that were condensed to code words/phrases, a small sampling of which is summarized as follows: value, human, resilience, impact, empathy (codes: finding meaning in work, resiliency, combating burnout, connecting with another human); block, course, opportunity, future, more (codes: incorporating into future courses, model for other blocks); real, lecture, learning, remember (codes: clinical correlation, real-world examples, practical application); positive, impact, career, interest (codes: positive examples/treatment outcomes, piquing interest in neurology). Themes were developed, revised, and finalized to demonstrate that Neuro Day helps to (1) re-energize medical education, (2) provide a humanistic element to learning, (3) serve as an educational model that can be adapted for other courses, (4) reinforce lecture content through patient interaction, and (5) positively change perceptions of what it means to be a neurologist (Table 2). These themes persisted across 2023 and 2024.

**Table 2:** Qualitative Analysis Demonstrating That Neuro Day Was Beneficial for the Medical Students for a Number of Different Thematic Reasons

Theme	Supporting quotation 2023	Supporting quotation 2024
Finding meaning in work	• Hearing the stories from patients about how much time doctors took to discuss their symptoms and include them in the plan for tests to perform and what treatments to pursue really re-oriented us to why we went to medical school in the first place • Very exciting and meaningful to hear from patients! • It shows the side of medicine we don't get to see much in first year • This day was so valuable and added a space to my mid-block burn out!	• I always appreciate an opportunity to zoom out and remember why it is I am doing what I am doing every day. Seeing the patients' success stories and their resilience was really refreshing • Not only did it help me see the real-life applications of the lesions we're learning in lecture, but it was an opportunity to focus on the patient care side of neurology, which is the reason many of us got into medicine • I think this was a great way to force students to take a step back, and realize why we are spending every day learning about these pathologies and mechanisms • This was possibly my favorite day of the whole school year
Humanizing medicine	• Loved the discussions of biopsychosocial aspects of care—so humanizing and refreshing • Neuro Day allowed me to assign a face and a story to a disease and see how it impacts a patient's life both at the onset of disease and through time • Neuro Day showed me the journeys that these patients, their families, and their physicians take while dealing with the disease including the frustrations when trying to come up with a diagnosis as well as the chronic aspects of certain diseases	• It was useful to consider how the conditions impacted the patients more holistically • My biggest take away from the NeuroPatient day was that every human being has a very different experience of life and that some are more fortunate than others. However, it was amazing to hear about the different ways that patients were able to cope with their conditions • I loved it. I think talking to patients directly is the best and most effective part of learning medicine • All of the speakers were so incredible, thoughtful, and fantastic at facilitating learning • I adored hearing the patient's stories and personal experiences
Adaptability of this educational technique for other courses	• This was a very interesting and fun experience, and I would like to have more of these in the future • Easily the best part of neurology so far. I hope this happens for every block! • I wish every course had a day like this!	• I wish all specialties/blocks did something like this • I wish there were more opportunities like this
Reinforcing course content	• Neuro day was a great experience and really made the lectures and content we have learned feel more “real” • It was very interesting to meet patients and hear their stories. It also helped enhance my understanding of clinical neurology and neuroanatomy • Great learning activity. Will make it easier to remember these conditions • Neuro Day was a great way for us to consolidate the information learned during our lectures and apply it to a real-life example	• Fun and helped solidify my learning and how these things manifest clinically • It is very easy to get lost in the details when strictly working with lecture material. Neuro day drove home a lot of big take away points for me • I very much appreciated connecting lecture topics to those living with neurological disorders. It really shined a light on the patient aspect of these diseases, and I believe it was a great supplement for student physicians
Changing perceptions about neurology	• It was amazing seeing how much of a positive impact some of the new medical advances can have on a patients such as deep brain stimulation for tremor • Neuro Day showed us [medical students] just how much a physician can have an impact on a patient's life through the care they provide	• This day gave me hope for a potential future in neurology • I came into Neuro without any interest in the specialty. I can honestly say that after the first block content, and even into the second block, my interest remained the same. However, seeing the ability to immediately use a physical exam and history to arrive at a diagnosis without prior imaging was empowering • My biggest takeaway is that I might actually be interested in pursuing neuro • I really enjoyed the interactive environment provided by Neuro Day, and it definitely piqued my interest into Neurology as a career • I had always heard that neurology was such a depressing field because you were basically just prolonging the inevitable. However, after neuro patient day, it was obvious that this is not true and that many neuro diseases/symptoms can at least be controlled • I was utterly blown away by the effect deep brain stimulation had on one patient's central tremor, which debunked the perception about neurology that there are not many positive outcomes • It was very fun to see how impactful a career in neurology can be, and how what we're learning plays a role in it

## Discussion and Lessons Learned

Neuro Day is an innovative in-person educational intervention that has proven to be feasible to implement on an annual basis for first-year medical students. The annual Neuro Day event has led to several beneficial outcomes, achieving most of the event objectives. First, Neuro Day has consistently led to increased interest in neurology and interest in pursuing neurology in the future. Second, Neuro Day plays an important role in reshaping perceptions about neurology, including improving comfort performing a neurologic examination and improving understanding of neurologic disease outcomes and treatment options. Specifically, Neuro Day showcased examples of positive treatment outcomes for patients, inspiring some students to consider a career in neurology, which they had not previously considered. These findings suggest that exposing students to patients with real neurologic conditions and hearing a range of experiences, including treatments that help patients to live their lives well, can help students to change their perception of what it means to care for patients with chronic neurologic disease. Third, students found case discussions, clinical skill demonstrations, and patient interactions to be the most helpful ways of learning neurology, all of which were innovative educational techniques used during Neuro Day. Fourth, the Neuro Day intervention may also play a role in reinforcing neuroscience lecture content and improving objective neurology knowledge. Fifth, Neuro Day may not only change student interests and perceptions but also change behavior as evidenced by the increase in the number of medical students pursuing a neurology externship between their first and second year of medical school. Furthermore, more students per year at our institution have been selecting a neurologic-related residency.

In addition to being a meaningful experience for medical students, patient-educators and physician-educators also expressed that this was an impactful experience, with patient-educators endorsing gratitude and physician-educators demonstrating increased professional fulfillment because of their participation. Patient-educators likely volunteered because of a preexisting interest in sharing their experiences with others.

Neurology is consistently ranked by medical students as one of the most, if not the most, difficult specialties.^4^ A systematic review found that trainees consistently report the lowest confidence in assessing, diagnosing, and treating patients with neurologic conditions.^4^ The reasons for these findings are often due to insufficient exposure and educational techniques. Innovative educational strategies to increase neurology accessibility include team-based learning, the use of online and virtual instructional techniques, mandating neurology training during the clinical years, integration of basic and clinical neurosciences during the preclinical years, the use of standardized patients to practice neurologic examination techniques, and development of a positive reputation for the neurologic field.^3,11,12^ The Neuro Day design combines several elements that have previously been shown to be effective for enhancing neurophilia, including the use of multimedia before Neuro Day to teach the neurologic examination, incorporation of real patients on Neuro Day, reinforcement of examination techniques that were shown in the pre–Neuro Day instructional video, reinforcement of concepts that were taught in lecture, using patient-educators as authentic teachers, and using physician-educators as positive examples of neurology mentors.

The use of video and multimedia has repeatedly led to sustained interest in neurology, better visualization of challenging concepts, and improved retention.^12-15^ E-learning became even more relevant during the COVID-19 pandemic. Indeed, telehealth communication enabled a virtual version of Neuro Day to proceed when social distancing prevented Neuro Day from occurring in person. Some studies have also developed and incorporated online gaming as part of the educational curriculum to entice medical students for further learning.^15^ However, it is important to note that the most successful strategies for improving neurology interest are often the simplest.^13^ In other words, making neurology simpler and more accessible to students is more important than making the learning activity extravagant or technologically savvy. Moreover, the importance of face-to-face interaction should not be minimized. Some of the most successful interventions to promote neurophilia have incorporated either standardized patients or patients with neurologic diagnoses.^11,12,16^

The placement in the curriculum during which educational interventions are introduced to trainees is also important. For programs that may not be able to offer a long-term third-year clinical clerkship, Neuro Day acts as an alternative or supplemental experience for trainees to gain exposure to clinical neurology early in their medical career. In addition, the Neuro Day model offers some flexibility in that it can be tailored to different levels of training and different curriculum time points. For example, a modified version of Neuro Day can be used to reinvigorate learning in neurology residents^17^ or can be used periodically to reinforce neurophilia throughout a clinician's medical career. Similarly, the Neuro Day design benefits both the intended recipient (medical students) and those providing the educational experience (patient-educators and physician-educators).

Another strength of this study is that not only were self-perceived knowledge, confidence, and comfort assessed, but objective measures were also analyzed. A limitation of other studies exploring neurology educational techniques is that they may only improve confidence in the material and not actually improve knowledge.^18^ A multiple-choice neurology quiz that was provided to medical students the day before and 2 days after Neuro Day demonstrated significant improvement in their objective clinical knowledge.

In addition, the number of students selecting a neurologic-based residency is growing at our institution. We have consistently filled the neurology residency fast-track slots with medical students from our own institution, and on average, more medical students per year have matched into a neurologic-based residency program from our institution in the years after Neuro Day was implemented. Of course, many factors may be related to these changes, including that the number of positions available and filling nationally has been steadily increasing over the years as well. While it is impossible to conclude that Neuro Day is directly responsible for the increased numbers of students pursuing a neurologic-related field from our institution, it is certainly an encouraging sign.

Neuro Day was implemented at a single center, so further research is needed to determine whether this educational intervention can be adapted and replicated at other institutions. The lack of a direct control group also makes it challenging to determine how much Neuro Day influences objective performance, including core neuroscience curriculum knowledge and beyond. In addition, when students were asked to rate their interest in future residency programs, only neurology was included of the 4 neurologic-related residency program choices, and it is possible that if child neurology, neurosurgery, or neurodevelopmental disabilities were listed as choices, additional students may have indicated interest. Future studies would be strengthened by evaluating national averages on examinations such as the National Board of Medical Examiners question banks and United States Medical Licensing Examination to determine whether objective neurology knowledge improves in programs that have dedicated and innovative neurology educational programs. Because Neuro Day involves direct interaction with patients, this may be a valuable time to formally assess medical student examination skills, interviewing skills, and communication skills. In the future, Neuro Day can be adapted to formally and objectively evaluate these skills in addition to objective knowledge.

There are several logistic challenges in designing Neuro Day, including coordinating multiple patients with neurologic diseases to attend for a 4-hour session, streamlining the groups of medical students to rotate from room to room, ensuring that media capabilities are accessible to demonstrate interesting imaging findings, and ensuring that enough physician-educators are available to be paired with patients. In spite of these logistic hurdles, Neuro Day has been successfully implemented for multiple years at our institution and has been consistently well received by all individuals involved.

Neuro Day is a feasible and sustainable educational intervention for medical students that has consistently led to increased interest in neurology, improved perceptions of neurology, and increased pursuance of a neurologic-related career. In addition, Neuro Day is a positive experience for patient-educators and physician-educators. The connections formed between patients, educators, and students as a result of Neuro Day can be a transformational experience for all involved.

## Glossary

COVID-19: coronavirus disease 2019
NRMP: National Resident Matching Program
PGY: postgraduate year
